# Supplementary material for: Healthcare Professionals’ Perspectives on HPV Recommendations: Themes of Interest to Different Population Groups and Strategies for Approaching Them
Source: Vaccines (Basel). 2024 Jul 6;12(7):748. doi: 10.3390/vaccines12070748 (PMC11281591; doi:10.3390/vaccines12070748)
Supplement: Supplementary file 1 [file vaccines-12-00748-s001.zip › Supplementary Material S1_SURVEY.pdf]

Identifying one-to-one communication themes on HPV and HPV vaccination

Welcome.

You have been invited to participate in a survey carried out in the framework of the European project PROTECT-EUROPE (EU4H-1, Project ID 101080046). PROTECT-EUROPE is an EU4Health Project that champions gender-neutral vaccination programme in EU Member States to provide protection for everyone against cancers caused by HPV e.g. cervical, anal, penile, vaginal, vulval and oropharyngeal.

What is the purpose of this study?

The aim of this study is to examine the factors that impact communication between doctors, young adolescents, parents, and caregivers when discussing HPV vaccination recommendations. We are interested in understanding how different topics are discussed based on patient characteristics. Specifically, we would like to explore the influence of factors such as religion, country of origin, educational level, and gender.

What does participating in this study involve?

We would like to invite you to participate in this study.

Your participation will involve completing a survey that should take approximately 10-15 minutes of your time. Your responses are extremely valuable as they will contribute to a comprehensive understanding of the communication issues surrounding HPV and aid in the development of appropriate interventions to improve this situation.

The database will be stored on FISABIO servers, but in case open access policies require the database to be placed in a repository it will always be anonymised.

Your participation in this survey is voluntary and you can withdraw at any time without any consequences. We thank you in advance for your time and dedication in completing the questionnaire.

The results will be available as part of deliverable 2.1 of Work Package 2: Best clinical practice: From science to delivery around the second quarter of 2024.

Your opinion is very important to us.

Thank you!

Do you agree to participate in this study?

- ◯ Yes
- ◯ No

Age

- ◯ 19-25
- ◯ 26-35
- ◯ 36-45
- ◯ 46-55
- ◯ 56-65
- ◯ 65+

Gender

◯ Man ◯ Woman ◯ Non-binary ◯ Other ◯ I don't want to answer

Country of origin

____

Medical speciality

- ◯ Family Doctor
- ◯ Paediatrician
- ◯ Nurse
- ◯ Other ______

Do you recommend the human papillomavirus (HPV) vaccine to the young adolescent patients you see in your practice?

○ Yes ○ No

What are the reasons why you do not recommend it?

- ☐ It is not compatible with my religious, ethical or moral beliefs.
- ☐ I believe it is not necessary to protect health.
- ☐ I consider that it can be dangerous for the patient's health.
- ☐ I prefer to prioritise other vaccines that I consider more appropriate for the adolescent.
- ☐ I don't trust this vaccine.
- ☐ It is not part of my routine tasks/competences.

Country of practice ______________________

This first block is about Religion. Religious beliefs shape perceptions of health, morality and medical decisions; the scientific literature highlights the influence of religion on opinions about the HPV vaccine.

To tailor the questions to your specific context, please indicate the religions of the individuals you encounter in your practice:

- ☐ Protestant
- ☐ Orthodoxy
- ☐ Catholicism
- ☐ Islam
- ☐ Hinduism
- ☐ Buddhism
- ☐ Judaism
- ☐ Other ______
- ☐ I do not provide care for any population with these characteristics.

Protestant:

Please indicate which of the following topics come up frequently during discussions about HPV and HPV vaccination with young adolescents and/or their parents or carers who identify with the Protestant faith.

- ☐ Taboos related to people's sexuality that may affect the conversation between the healthcare professional and the adolescent or his/her parents/guardians.
- ☐ Negative perceptions of the HPV vaccine in their environment (e.g. that it encourages promiscuity) and how this perception influences their decision to be vaccinated (or not).
- ☐ Misinformation about the HPV vaccine, such as lack of efficacy, safety or unproven adverse effects (e.g. that it causes infertility).
- ☐ Considerations about the lack of benefit of the vaccine at the time, HPV being a sexually transmitted infection and given that it is administered at a very young age.
- ☐ Difficulties in accessing the health system and completing the recommended schedules (2 or 3 doses).
- ☐ When offering the vaccine to a male, the false belief that it only has health benefits for men who have sex with men.
- ☐ Lack of knowledge about HPV infection and its consequences.
- ☐ None.
- ☐ Don't know.

Orthodoxy:

Please indicate which of the following topics come up frequently during discussions about HPV and HPV vaccination with young adolescents and/or their parents or carers who identify with the Orthodox faith

- ☐ Taboos related to people's sexuality that may affect the conversation between the healthcare professional and the adolescent or his/her parents/guardians.
- ☐ Negative perceptions of the HPV vaccine in their environment (e.g. that it encourages promiscuity) and how this perception influences their decision to be vaccinated (or not).
- ☐ Misinformation about the HPV vaccine, such as lack of efficacy, safety or unproven adverse effects (e.g. that it causes infertility).
- ☐ Considerations about the lack of benefit of the vaccine at the time, HPV being a sexually transmitted infection and given that it is administered at a very young age.
- ☐ Difficulties in accessing the health system and completing the recommended schedules (2 or 3 doses).
- ☐ When offering the vaccine to a male, the false belief that it only has health benefits for men who have sex with men.
- ☐ Lack of knowledge about HPV infection and its consequences.
- ☐ None.
- ☐ Don't know.

Catholicism:

Please indicate which of the following topics come up frequently during discussions about HPV and HPV vaccination with young adolescents and/or their parents or carers who identify with the Catholic faith

- ☐ Taboos related to people's sexuality that may affect the conversation between the healthcare professional and the adolescent or his/her parents/guardians.
- ☐ Negative perceptions of the HPV vaccine in their environment (e.g. that it encourages promiscuity) and how this perception influences their decision to be vaccinated (or not).
- ☐ Misinformation about the HPV vaccine, such as lack of efficacy, safety or unproven adverse effects (e.g. that it causes infertility).
- ☐ Considerations about the lack of benefit of the vaccine at the time, HPV being a sexually transmitted infection and given that it is administered at a very young age.
- ☐ Difficulties in accessing the health system and completing the recommended schedules (2 or 3 doses).
- ☐ When offering the vaccine to a male, the false belief that it only has health benefits for men who have sex with men.
- ☐ Lack of knowledge about HPV infection and its consequences.
- ☐ None.
- ☐ Don't know.

Islam:

Please indicate which of the following topics come up frequently during discussions about HPV and HPV vaccination with young adolescents and/or their parents or carers who identify with the Islamic faith

- ☐ Taboos related to people's sexuality that may affect the conversation between the healthcare professional and the adolescent or his/her parents/guardians.
- ☐ Negative perceptions of the HPV vaccine in their environment (e.g. that it encourages promiscuity) and how this perception influences their decision to be vaccinated (or not).
- ☐ Misinformation about the HPV vaccine, such as lack of efficacy, safety or unproven adverse effects (e.g. that it causes infertility).
- ☐ Considerations about the lack of benefit of the vaccine at the time, HPV being a sexually transmitted infection and given that it is administered at a very young age.
- ☐ Difficulties in accessing the health system and completing the recommended schedules (2 or 3 doses).
- ☐ When offering the vaccine to a male, the false belief that it only has health benefits for men who have sex with men.
- ☐ Lack of knowledge about HPV infection and its consequences.
- ☐ None.
- ☐ Don't know.

Hinduism:

Please indicate which of the following topics come up frequently during discussions about HPV and HPV vaccination with young adolescents and/or their parents or carers who identify with the Hindu faith

- ☐ Taboos related to people's sexuality that may affect the conversation between the healthcare professional and the adolescent or his/her parents/guardians.
- ☐ Negative perceptions of the HPV vaccine in their environment (e.g. that it encourages promiscuity) and how this perception influences their decision to be vaccinated (or not).
- ☐ Misinformation about the HPV vaccine, such as lack of efficacy, safety or unproven adverse effects (e.g. that it causes infertility).
- ☐ Considerations about the lack of benefit of the vaccine at the time, HPV being a sexually transmitted infection and given that it is administered at a very young age.
- ☐ Difficulties in accessing the health system and completing the recommended schedules (2 or 3 doses).
- ☐ When offering the vaccine to a male, the false belief that it only has health benefits for men who have sex with men.
- ☐ Lack of knowledge about HPV infection and its consequences.
- ☐ None.
- ☐ Don't know.

Buddhism:

Please indicate which of the following topics come up frequently during discussions about HPV and HPV vaccination with young adolescents and/or their parents or carers who identify with the Buddhist confession

- ☐ Taboos related to people's sexuality that may affect the conversation between the healthcare professional and the adolescent or his/her parents/guardians.
- ☐ Negative perceptions of the HPV vaccine in their environment (e.g. that it encourages promiscuity) and how this perception influences their decision to be vaccinated (or not).
- ☐ Misinformation about the HPV vaccine, such as lack of efficacy, safety or unproven adverse effects (e.g. that it causes infertility).
- ☐ Considerations about the lack of benefit of the vaccine at the time, HPV being a sexually transmitted infection and given that it is administered at a very young age.
- ☐ Difficulties in accessing the health system and completing the recommended schedules (2 or 3 doses).
- ☐ When offering the vaccine to a male, the false belief that it only has health benefits for men who have sex with men.
- ☐ Lack of knowledge about HPV infection and its consequences.
- ☐ None.
- ☐ Don't know.

Judaism:

Please indicate which of the following topics come up frequently during discussions about HPV and HPV vaccination with young adolescents and/or their parents or carers who identify with the Jewish faiths

- ☐ Taboos related to people's sexuality that may affect the conversation between the healthcare professional and the adolescent or his/her parents/guardians.
- ☐ Negative perceptions of the HPV vaccine in their environment (e.g. that it encourages promiscuity) and how this perception influences their decision to be vaccinated (or not).
- ☐ Misinformation about the HPV vaccine, such as lack of efficacy, safety or unproven adverse effects (e.g. that it causes infertility).
- ☐ Considerations about the lack of benefit of the vaccine at the time, HPV being a sexually transmitted infection and given that it is administered at a very young age.
- ☐ Difficulties in accessing the health system and completing the recommended schedules (2 or 3 doses).
- ☐ When offering the vaccine to a male, the false belief that it only has health benefits for men who have sex with men.
- ☐ Lack of knowledge about HPV infection and its consequences.
- ☐ None.
- ☐ Don't know.

Other: Taking into account the religion that you specified as other.

Please indicate which of the following topics come up frequently during discussions about HPV and HPV vaccination with young adolescents and/or their parents or carers who identify with that religious faiths

- ☐ Taboos related to people's sexuality that may affect the conversation between the healthcare professional and the adolescent or his/her parents/guardians.
- ☐ Negative perceptions of the HPV vaccine in their environment (e.g. that it encourages promiscuity) and how this perception influences their decision to be vaccinated (or not).
- ☐ Misinformation about the HPV vaccine, such as lack of efficacy, safety or unproven adverse effects (e.g. that it causes infertility).
- ☐ Considerations about the lack of benefit of the vaccine at the time, HPV being a sexually transmitted infection and given that it is administered at a very young age.
- ☐ Difficulties in accessing the health system and completing the recommended schedules (2 or 3 doses).
- ☐ When offering the vaccine to a male, the false belief that it only has health benefits for men who have sex with men.
- ☐ Lack of knowledge about HPV infection and its consequences.
- ☐ None.
- ☐ Don't know.

Do you think there are any issues that come up recurrently in conversation with people of any religion that have not been mentioned before?

◯ Yes ◯ No

Please describe the topic/issue and the population you think it affects.

This block deals with region of origin of immigrants and descendants of immigrants, ethnicity and/or legal status in the country. The influence of country of origin or ethnicity on opinions about HPV vaccination is a relevant issue in the scientific literature. Studies have shown that factors such as barriers to access, misinformation and cultural concerns can affect vaccine acceptance in different communities.
Undocumented immigrant status may also influence habits and opinions about HPV vaccination. Barriers and challenges associated with their status may affect HPV vaccine uptake and access.

Do you provide medical services in your practice to immigrant populations or descendants of immigrants from the following regions of origin, ethnic backgrounds, or individuals with irregular immigration status in the country?

- ☐ Other European (excluding the country's population)
- ☐ Near or Middle East (Turkey, Syria, Jordan, Egypt, Pakistan, Iran, etc.)
- ☐ Far East (China, Korea, Japan, Philippines, Thailand, etc.)
- ☐ North African
- ☐ Sub-Saharan Africa
- ☐ Latin America
- ☐ Rest of America
- ☐ Gypsies, Roma and Travellers (GRT)
- ☐ Undocumented migrant population
- ☐ I do not provide care for any population with these characteristics.

Other European country:

Please indicate which of the following topics frequently come up during discussions about HPV and HPV vaccination with young adolescents and/or their parents/caregivers from Other European country

- ☐ Taboos related to people's sexuality that may affect the conversation between the healthcare professional and the adolescent or his/her parents/guardians.
- ☐ Negative perceptions of the HPV vaccine in their environment (e.g. that it encourages promiscuity) and how this perception influences their decision to be vaccinated (or not).
- ☐ Misinformation about the HPV vaccine, such as lack of efficacy, safety or unproven adverse effects (e.g. that it causes infertility).
- ☐ Considerations about the lack of benefit of the vaccine at the time, HPV being a sexually transmitted infection and given that it is administered at a very young age.
- ☐ Difficulties in accessing the health system and completing the recommended schedules (2 or 3 doses).
- ☐ When offering the vaccine to a male, the false belief that it only has health benefits for men who have sex with men.
- ☐ Lack of knowledge about HPV infection and its consequences.
- ☐ None.
- ☐ Don't know.

Near or Middle East:

Please indicate which of the following topics frequently come up during discussions about HPV and HPV vaccination with young adolescents and/or their parents/caregivers from Near or Middle East

- ☐ Taboos related to people's sexuality that may affect the conversation between the healthcare professional and the adolescent or his/her parents/guardians.
- ☐ Negative perceptions of the HPV vaccine in their environment (e.g. that it encourages promiscuity) and how this perception influences their decision to be vaccinated (or not).
- ☐ Misinformation about the HPV vaccine, such as lack of efficacy, safety or unproven adverse effects (e.g. that it causes infertility).
- ☐ Considerations about the lack of benefit of the vaccine at the time, HPV being a sexually transmitted infection and given that it is administered at a very young age.
- ☐ Difficulties in accessing the health system and completing the recommended schedules (2 or 3 doses).
- ☐ When offering the vaccine to a male, the false belief that it only has health benefits for men who have sex with men.
- ☐ Lack of knowledge about HPV infection and its consequences.
- ☐ None.
- ☐ Don't know.

Far East:

Please indicate which of the following topics frequently come up during discussions about HPV and HPV vaccination with young adolescents and/or their parents/caregivers from Far East

☐ Taboos related to people's sexuality that may affect the conversation between the healthcare professional and the adolescent or his/her parents/guardians.
☐ Negative perceptions of the HPV vaccine in their environment (e.g. that it encourages promiscuity) and how this perception influences their decision to be vaccinated (or not).
☐ Misinformation about the HPV vaccine, such as lack of efficacy, safety or unproven adverse effects (e.g. that it causes infertility).
☐ Considerations about the lack of benefit of the vaccine at the time, HPV being a sexually transmitted infection and given that it is administered at a very young age.
☐ Difficulties in accessing the health system and completing the recommended schedules (2 or 3 doses).
☐ When offering the vaccine to a male, the false belief that it only has health benefits for men who have sex with men.
☐ Lack of knowledge about HPV infection and its consequences.
☐ None.
☐ Don't know.

North Africa:

Please indicate which of the following topics frequently come up during discussions about HPV and HPV vaccination with young adolescents and/or their parents/caregivers from North Africa

☐ Taboos related to people's sexuality that may affect the conversation between the healthcare professional and the adolescent or his/her parents/guardians.
☐ Negative perceptions of the HPV vaccine in their environment (e.g. that it encourages promiscuity) and how this perception influences their decision to be vaccinated (or not).
☐ Misinformation about the HPV vaccine, such as lack of efficacy, safety or unproven adverse effects (e.g. that it causes infertility).
☐ Considerations about the lack of benefit of the vaccine at the time, HPV being a sexually transmitted infection and given that it is administered at a very young age.
☐ Difficulties in accessing the health system and completing the recommended schedules (2 or 3 doses).
☐ When offering the vaccine to a male, the false belief that it only has health benefits for men who have sex with men.
☐ Lack of knowledge about HPV infection and its consequences.
☐ None.
☐ Don't know.

Sub-Saharan Africa:

Please indicate which of the following topics frequently come up during discussions about HPV and HPV vaccination with young adolescents and/or their parents/caregivers from Sub-Saharan Africa

☐ Taboos related to people's sexuality that may affect the conversation between the healthcare professional and the adolescent or his/her parents/guardians.
☐ Negative perceptions of the HPV vaccine in their environment (e.g. that it encourages promiscuity) and how this perception influences their decision to be vaccinated (or not).
☐ Misinformation about the HPV vaccine, such as lack of efficacy, safety or unproven adverse effects (e.g. that it causes infertility).
☐ Considerations about the lack of benefit of the vaccine at the time, HPV being a sexually transmitted infection and given that it is administered at a very young age.
☐ Difficulties in accessing the health system and completing the recommended schedules (2 or 3 doses).
☐ When offering the vaccine to a male, the false belief that it only has health benefits for men who have sex with men.
☐ Lack of knowledge about HPV infection and its consequences.
☐ None.
☐ Don't know.

Latin America:

Please indicate which of the following topics frequently come up during discussions about HPV and HPV vaccination with young adolescents and/or their parents/caregivers from Latin America

- ☐ Taboos related to people's sexuality that may affect the conversation between the healthcare professional and the adolescent or his/her parents/guardians.
- ☐ Negative perceptions of the HPV vaccine in their environment (e.g. that it encourages promiscuity) and how this perception influences their decision to be vaccinated (or not).
- ☐ Misinformation about the HPV vaccine, such as lack of efficacy, safety or unproven adverse effects (e.g. that it causes infertility).
- ☐ Considerations about the lack of benefit of the vaccine at the time, HPV being a sexually transmitted infection and given that it is administered at a very young age.
- ☐ Difficulties in accessing the health system and completing the recommended schedules (2 or 3 doses).
- ☐ When offering the vaccine to a male, the false belief that it only has health benefits for men who have sex with men.
- ☐ Lack of knowledge about HPV infection and its consequences.
- ☐ None.
- ☐ Don't know.

From Rest of America:

Please indicate which of the following topics frequently come up during discussions about HPV and HPV vaccination with young adolescents and/or their parents/caregivers from Rest of America

- ☐ Taboos related to people's sexuality that may affect the conversation between the healthcare professional and the adolescent or his/her parents/guardians.
- ☐ Negative perceptions of the HPV vaccine in their environment (e.g. that it encourages promiscuity) and how this perception influences their decision to be vaccinated (or not).
- ☐ Misinformation about the HPV vaccine, such as lack of efficacy, safety or unproven adverse effects (e.g. that it causes infertility).
- ☐ Considerations about the lack of benefit of the vaccine at the time, HPV being a sexually transmitted infection and given that it is administered at a very young age.
- ☐ Difficulties in accessing the health system and completing the recommended schedules (2 or 3 doses).
- ☐ When offering the vaccine to a male, the false belief that it only has health benefits for men who have sex with men.
- ☐ Lack of knowledge about HPV infection and its consequences.
- ☐ None.
- ☐ Don't know.

Gypsies, Roma or Travellers (GRT):

Please indicate which of the following topics frequently arise during discussions about HPV and HPV vaccination with young adolescents and/or their parents/caregivers of Roma, Gypsy, Traveller (GRT) ethnicity

- ☐ Taboos related to people's sexuality that may affect the conversation between the healthcare professional and the adolescent or his/her parents/guardians.
- ☐ Negative perceptions of the HPV vaccine in their environment (e.g. that it encourages promiscuity) and how this perception influences their decision to be vaccinated (or not).
- ☐ Misinformation about the HPV vaccine, such as lack of efficacy, safety or unproven adverse effects (e.g. that it causes infertility).
- ☐ Considerations about the lack of benefit of the vaccine at the time, HPV being a sexually transmitted infection and given that it is administered at a very young age.
- ☐ Difficulties in accessing the health system and completing the recommended schedules (2 or 3 doses).
- ☐ When offering the vaccine to a male, the false belief that it only has health benefits for men who have sex with men.
- ☐ Lack of knowledge about HPV infection and its consequences.
- ☐ None.
- ☐ Don't know.

Undocumented migrant population:

Please indicate which of the following topics frequently arise during discussions about HPV and HPV vaccination with young adolescents and/or their parents/caregivers with Undocumented migrant status

- ☐ Taboos related to people's sexuality that may affect the conversation between the healthcare professional and the adolescent or his/her parents/guardians.
- ☐ Negative perceptions of the HPV vaccine in their environment (e.g. that it encourages promiscuity) and how this perception influences their decision to be vaccinated (or not).
- ☐ Misinformation about the HPV vaccine, such as lack of efficacy, safety or unproven adverse effects (e.g. that it causes infertility).
- ☐ Considerations about the lack of benefit of the vaccine at the time, HPV being a sexually transmitted infection and given that it is administered at a very young age.
- ☐ Difficulties in accessing the health system and completing the recommended schedules (2 or 3 doses).
- ☐ When offering the vaccine to a male, the false belief that it only has health benefits for men who have sex with men.
- ☐ Lack of knowledge about HPV infection and its consequences.
- ☐ None.
- ☐ Don't know.

Do you think there are any issue that come up more often people of a particular region of origin or ethnicity and that have not been mentioned above?

◯ Yes ◯ No

Please describe the topic/issue and the population you think it affects.

This block is about Gender (of the young adolescent eligible for vaccination). Gender plays a crucial role in physician-patient communication about Human Papillomavirus (HPV). The relevance of considering gender lies in the fact that HPV affects both men and women, but in a differentiated manner. It is essential to understand how gender roles, cultural norms and social expectations can influence communication about HPV. These differences can affect risk perception, attitudes toward the vaccine, and preventive behaviors.

What are the genders of the young adolescents you see in your medical practice?

- ☐ Boys.
- ☐ Girls.
- ☐ Other ______
- ☐ I do not provide care for any young adolescents.

Boys and/or their parents/carers:

Indicate which of the following topics often come up in discussions about HPV and HPV vaccination with Boys and/or their parents/carers

- ☐ Taboos related to people's sexuality that may affect the conversation between the healthcare professional and the adolescent or his/her parents/guardians.
- ☐ Negative perceptions of the HPV vaccine in their environment (e.g. that it encourages promiscuity) and how this perception influences their decision to be vaccinated (or not).
- ☐ Misinformation about the HPV vaccine, such as lack of efficacy, safety or unproven adverse effects (e.g. that it causes infertility).
- ☐ Considerations about the lack of benefit of the vaccine at the time, HPV being a sexually transmitted infection and given that it is administered at a very young age.
- ☐ Difficulties in accessing the health system and completing the recommended schedules (2 or 3 doses).
- ☐ When offering the vaccine to a male, the false belief that it only has health benefits for men who have sex with men.
- ☐ Lack of knowledge about HPV infection and its consequences.
- ☐ None.
- ☐ Don't know.

Girls and/or their parents/carers:

Indicate which of the following topics often come up in discussions about HPV and HPV vaccination with Girls and/or their parents/carers

- ☐ Taboos related to people's sexuality that may affect the conversation between the healthcare professional and the adolescent or his/her parents/guardians.
- ☐ Negative perceptions of the HPV vaccine in their environment (e.g. that it encourages promiscuity) and how this perception influences their decision to be vaccinated (or not).
- ☐ Misinformation about the HPV vaccine, such as lack of efficacy, safety or unproven adverse effects (e.g. that it causes infertility).
- ☐ Considerations about the lack of benefit of the vaccine at the time, HPV being a sexually transmitted infection and given that it is administered at a very young age.
- ☐ Difficulties in accessing the health system and completing the recommended schedules (2 or 3 doses).
- ☐ When offering the vaccine to a male, the false belief that it only has health benefits for men who have sex with men.
- ☐ Lack of knowledge about HPV infection and its consequences.
- ☐ None.
- ☐ Don't know.

Other:

Indicate which of the following topics often come up in discussions about HPV and HPV vaccination with young adolescents with a gender other than boys or girls and/or with their parents/carers

- ☐ Taboos related to people's sexuality that may affect the conversation between the healthcare professional and the adolescent or his/her parents/guardians.
- ☐ Negative perceptions of the HPV vaccine in their environment (e.g. that it encourages promiscuity) and how this perception influences their decision to be vaccinated (or not).
- ☐ Misinformation about the HPV vaccine, such as lack of efficacy, safety or unproven adverse effects (e.g. that it causes infertility).
- ☐ Considerations about the lack of benefit of the vaccine at the time, HPV being a sexually transmitted infection and given that it is administered at a very young age.
- ☐ Difficulties in accessing the health system and completing the recommended schedules (2 or 3 doses).
- ☐ When offering the vaccine to a male, the false belief that it only has health benefits for men who have sex with men.
- ☐ Lack of knowledge about HPV infection and its consequences.
- ☐ None.
- ☐ Don't know.

Do you think there are any issues that come up recurrently in conversation with people of a particular gender and that have not been mentioned above?

○ Yes ○ No

Please describe the topic/issue and the population you think it affects.

This block deals with Educational level and language proficency. Educational level and language proficiency are key elements in doctor-patient communication about HPV vaccination. These factors can influence the understanding of medical information and make it difficult to make informed decisions. Therefore, it is essential to tailor communication with these aspects in mind to promote better understanding and acceptance of the HPV vaccine.

Which of the following groups do you provide medical services to, based on their educational level and their proficiency in the local language?

- ☐ Low level of education (incomplete school education)
- ☐ Medium level of education (completed school education or vocational training)
- ☐ High level of education (university or higher education)
- ☐ Low proficiency in the local language
- ☐ I do not provide care for any population with these characteristics.

Low level of education (incomplete school education):

Indicate which of the following topics often come up in discussions about HPV and HPV vaccination with Low level of education (incomplete school education)

- ☐ Taboos related to people's sexuality that may affect the conversation between the healthcare professional and the adolescent or his/her parents/guardians.
- ☐ Negative perceptions of the HPV vaccine in their environment (e.g. that it encourages promiscuity) and how this perception influences their decision to be vaccinated (or not).
- ☐ Misinformation about the HPV vaccine, such as lack of efficacy, safety or unproven adverse effects (e.g. that it causes infertility).
- ☐ Considerations about the lack of benefit of the vaccine at the time, HPV being a sexually transmitted infection and given that it is administered at a very young age.
- ☐ Difficulties in accessing the health system and completing the recommended schedules (2 or 3 doses).
- ☐ When offering the vaccine to a male, the false belief that it only has health benefits for men who have sex with men.
- ☐ Lack of knowledge about HPV infection and its consequences.
- ☐ None.
- ☐ Don't know.

Medium level of education (completed school education or vocational training):

Indicate which of the following topics often come up in discussions about HPV and HPV vaccination with Medium level of education (completed school education or vocational training)

- ☐ Taboos related to people's sexuality that may affect the conversation between the healthcare professional and the adolescent or his/her parents/guardians.
- ☐ Negative perceptions of the HPV vaccine in their environment (e.g. that it encourages promiscuity) and how this perception influences their decision to be vaccinated (or not).
- ☐ Misinformation about the HPV vaccine, such as lack of efficacy, safety or unproven adverse effects (e.g. that it causes infertility).
- ☐ Considerations about the lack of benefit of the vaccine at the time, HPV being a sexually transmitted infection and given that it is administered at a very young age.
- ☐ Difficulties in accessing the health system and completing the recommended schedules (2 or 3 doses).
- ☐ When offering the vaccine to a male, the false belief that it only has health benefits for men who have sex with men.
- ☐ Lack of knowledge about HPV infection and its consequences.
- ☐ None.
- ☐ Don't know.

High level of education (university or higher education):

Indicate which of the following topics often come up in discussions about HPV and HPV vaccination with High level of education (university or higher education)

- ☐ Taboos related to people's sexuality that may affect the conversation between the healthcare professional and the adolescent or his/her parents/guardians.
- ☐ Negative perceptions of the HPV vaccine in their environment (e.g. that it encourages promiscuity) and how this perception influences their decision to be vaccinated (or not).
- ☐ Misinformation about the HPV vaccine, such as lack of efficacy, safety or unproven adverse effects (e.g. that it causes infertility).
- ☐ Considerations about the lack of benefit of the vaccine at the time, HPV being a sexually transmitted infection and given that it is administered at a very young age.
- ☐ Difficulties in accessing the health system and completing the recommended schedules (2 or 3 doses).
- ☐ When offering the vaccine to a male, the false belief that it only has health benefits for men who have sex with men.
- ☐ Lack of knowledge about HPV infection and its consequences.
- ☐ None.
- ☐ Don't know.

Low proficiency in the local language:

Indicate which of the following topics often come up in discussions about HPV and HPV vaccination with Low proficiency in the local language

- ☐ Taboos related to people's sexuality that may affect the conversation between the healthcare professional and the adolescent or his/her parents/guardians.
- ☐ Negative perceptions of the HPV vaccine in their environment (e.g. that it encourages promiscuity) and how this perception influences their decision to be vaccinated (or not).
- ☐ Misinformation about the HPV vaccine, such as lack of efficacy, safety or unproven adverse effects (e.g. that it causes infertility).
- ☐ Considerations about the lack of benefit of the vaccine at the time, HPV being a sexually transmitted infection and given that it is administered at a very young age.
- ☐ Difficulties in accessing the health system and completing the recommended schedules (2 or 3 doses).
- ☐ When offering the vaccine to a male, the false belief that it only has health benefits for men who have sex with men.
- ☐ Lack of knowledge about HPV infection and its consequences.
- ☐ None.
- ☐ Don't know.

Do you think there are any issues that come up recurrently in conversation with people of a particular educational level and that have not been mentioned above?

◯ Yes ◯ No

Please describe the topic/issue and the population you think it affects.

Proposals to improve one-to-one communication

We would now like to ask you if you have any suggestions for overcoming communication problems that may arise with the young adolescents and/or their parents/carer when offering the HPV vaccine. Please indicate the characteristics of the population for which you have suggestions to improve communication:

- ☐ Religion.
- ☐ Region of origin of immigrants and descendants of immigrants, ethnicity and/or legal status in the country.
- ☐ Gender (of the young adolescents eligible for vaccination).
- ☐ Educational level and language proficency (of the parent or carer of the young adolescent eligible for vaccination).
- ☐ Other
- ☐ None

Please let us know your suggestions for improving communication about HPV and HPV vaccination with young adolescent and/or their parents/carers based on their religion. Please indicate the religion(s) about which you are making the recommendation.

Please let us know your suggestions for improving communication about HPV and HPV vaccination with young adolescent and/or their parents/carers based on their region of origin of immigrants and descendants of immigrants, ethnicity and/or legal status in the country. Please indicate the region of origin, ethnicity and/or legal status in the country for which you are making the recommendation.

Please let us know your suggestions for improving communication about HPV and HPV vaccination with young adolescent and/or their parents/carers based on the gender of the young adolescent eligible for vaccination. Please indicate the gender(s) about which you are making the recommendation.

Please let us know your suggestions for improving communication about HPV and HPV vaccination with young adolescent and/or their parents/carers based on their educational level and/or language proficency. Please indicate the educational level and/or language proficency about which you are making the recommendation.

Please provide us with suggestions for improving communication about HPV and HPV vaccination with young adolescent and/or their parents/carers based on other personal characteristics than those identified.

Do you know of any guidelines, strategies or training materials on how to improve doctor-patient communication taking into account patient characteristics (gender, ethnicity, religion, low health literacy, etc.)?
Please could you provide us with their reference or send us the document to the following address: jaime.fons@fisabio.es

We are interested in conducting a second study to deepen our understanding of communication strategies to lead with these topics in the mentioned populations. If you are willing to participate, please send us an e-mail to the following address: jaime.fons@fisabio.es

Thank you very much for your participation
