# Supplementary material for: Healthcare Professionals’ Perspectives on HPV Recommendations: Themes of Interest to Different Population Groups and Strategies for Approaching Them
Source: Vaccines (Basel). 2024 Jul 6;12(7):748. doi: 10.3390/vaccines12070748 (PMC11281591; doi:10.3390/vaccines12070748)
Supplement: Supplementary file 1 [file vaccines-12-00748-s001.zip › Supplementary Material S2_Table.pdf]

Supplementary Material 2. Proportion of respondents serving each population who consider that this theme comes up during discussions recommending HPV vaccination (multiple-response answers).

		Moral/Cultural			Informational				
		**A. Taboos**	**B. N-percep**	**C. Lack benef**	**D. Misinfo**	**E. Lack known**	**F. MV**	**G. Access**	**None**
		% (n)	% (n)	% (n)	% (n)	% (n)	% (n)	% (n)	% (n)
Religion	PROT	16.7 (3)	16.7 (3)	11.1 (2)	27.8 (5)	44.4 (8)	0.0 (0)	0.0 (0)	16.7 (3)
	ORTH	26.3 (5)	31.6 (6)	21.1 (4)	42.1 (8)	31.6 (6)	10.5 (2)	5.3 (1)	15.8 (3)
	CATH	26.0 (52)	39.0 (78)	29.5 (59)	42.5 (85)	48.5 (97)	19.5 (39)	3.5 (7)	10.5 (21)
	ISLM	43.3 (39)	28.9 (26)	21.1 (19)	33.3 (30)	58.9 (53)	22.2 (20)	14.4 (13)	13.3 (12)
	HIN	26.9 (7)	19.2 (5)	15.4 (4)	23.1 (6)	42.3 (11)	11.5 (3)	7.7 (2)	23.1 (6)
Region of origin and/or legal status in the country	OE	19.4 (25)	24.8 (32)	25.6 (33)	37.21 (48)	49.6 (64)	13.9 (18)	11.6 (15)	16.3 (21)
	NOME	34.8 (24)	29.0 (20)	29.0 (20)	33.3 (23)	55.1 (38)	21.7 (15)	18.8 (13)	14.5 (10)
	FE	25.0 (13)	11.5 (6)	17.3 (9)	25.0 (13)	40.4 (21)	13.5 (7)	9.6 (5)	28.9 (15)
	NA	39.1 (25)	20.3 (13)	20.3 (13)	29.7 (19)	59.4 (38)	17.12 (11)	21.9 (14)	12.5 (8)
	SSA	41.7 (15)	16.7 (6)	19.4 (7)	30.6 (11)	44.4 (16)	16.7 (6)	25.0 (9)	19.4 (7)
	LA	19.7 (13)	10.6 (7)	10.6 (7)	28.8 (19)	40.9 (27)	16.7 (11)	13.6 (9)	24.2 (16)
	ROA	20.0 (3)	13.3 (2)	13.3 (2)	26.7 (4)	40.0 (6)	13.3 (2)	0.0 (0)	20.0 (3)
	GRT	32.7 (16)	28.6 (14)	28.6 (14)	36.7 (18)	53.1 (26)	20.4 (10)	28.6 (14)	12.2 (6)
	UNDOC	35.1 (13)	18.9 (7)	27.0 (10)	29.7 (11)	48.7 (18)	8.1 (3)	27.0 (10)	2.7 (1)
Minor's gender	BOYS	22.1 (43)	22.6 (44)	30.3 (59)	41.5 (81)	58.5 (114)	28.2 (55)	8.7 (17)	6.7 (13)
	GIRLS	28.6 (58)	38.4 (78)	34 (69)	45.3 (92)	52.2 (106)	9.9 (20)	6.9 (14)	9.9 (20)
Language level and proficiency	LLE	31.7 (39)	33.3 (41)	32.5 (40)	39.8 (49)	65.0 (80)	19.5 (24)	13.8 (17)	4.1 (5)
	MLE	27.4 (46)	37.5 (63)	33.9 (57)	46.4 (78)	51.2 (86)	19.1 (32)	7.7 (13)	3.6 (6)
	HLE	20.0 (26)	30.0 (39)	35.4 (46)	43.1 (56)	38.5 (50)	14.6 (19)	3.9 (5)	13.9 (18)
	LPLL	35.3 (24)	29.4 (20)	26.5 (18)	33.8 (23)	61.8 (42)	16.2 (11)	20.6 (14)	8.8 (6)

Themes: A. Taboos related to people's sexuality that may affect the conversation between a healthcare professional and an adolescent or his/her parents/guardians (A. Taboos); B. Negative perceptions of the HPVV in their environment (e.g., that it encourages promiscuity) and how this perception influences their decision to be vaccinated (or not) (B. N-percep); C. Considerations about the lack of benefit of the vaccine at the time, HPV being a sexually transmitted infection and given that it is administered at a very young age (C. Lack benef); D. Misinformation about the HPVV, such as lack of efficacy and safety or unproven adverse effects (e.g., that it causes infertility) (D. Misinfo); E. Lack of knowledge about HPV infection and its consequences (E. Lack known); F. When offering the vaccine to a male, the false belief that it only has health benefits for men who have sex with men (F. MV); G. Difficulties in accessing the health system and completing the recommended schedules (2 or 3 doses) (G. Access). Religion: Protestantism (PROT; N = 18), Orthodoxy (ORTH; N = 19), Catholicism (CATH; N = 200), Islam (ISLM; N = 90), Hinduism (HIN; N = 26); Region of origin of immigrants and descendants of immigrants, ethnicity and/or legal status in the country: Other European (excluding the country's population) (OE; N = 129), the Near or Middle East (Turkey, Syria, Jordan, Egypt, Pakistan, Iran, etc.) (NOME; N = 69), the Far East (China, Korea, Japan, Philippines, Thailand, etc.) (FE; N = 52), North African (NA; N = 64), Sub-Saharan Africa (SSA; N = 36), Latin America (LA; N = 66), the rest of America (ROA; N = 15), Gypsies, Roma and Travellers (GRT; N = 49), undocumented migrant population (UNDOC; N = 37); Gender: Boys (N = 195), girls (N = 203); Educational level and language proficiency: Low level of education (incomplete school education) (LLE; N = 123), medium level of education (completed school education or vocational training) (MLE; N = 168), high level of education (university or higher education) (HLE; N = 68), low proficiency in the local language (LPLL; N = 68).
